# A Survey of Public Funding of Cancer Research in the European Union

**DOI:** 10.1371/journal.pmed.0030267

**Published:** 2006-07-18

**Authors:** Seth Eckhouse, Richard Sullivan

## Abstract

The European Cancer Research Funding Survey found inadequate public funding of cancer research, say Sullivan and Eckhouse.

In 2002 cancer accounted for 16.7% of the total burden of disease in the European Union (EU), compared with 25.3% and 17.1% for mental health and cardiovascular diseases, respectively [ 1]. In the period relevant to this Policy Forum, cancer was the second largest cause of mortality in the EU, accounting for 27.08% of all deaths. With the ageing population and the continuing impact of tobacco-associated cancers, it is predicted that cancer rates could increase by 50% to 15 million new cases worldwide in the year 2020 [ 1].

A recent update of cancer incidence and mortality for the EU in 2004 by the International Agency for Research on Cancer estimated 2,886,800 incident cases of cancer diagnosed and 1,711,000 cancer deaths [ 2]. As the authors of the update note, “the ageing of the European population will cause these numbers to continue to increase even if age-specific rates remain constant.” Furthermore, despite substantial progress through public health measures (i.e., tobacco control) and new treatments, survival is still poor for many cancers, such as lung cancer. These figures speak for themselves in making the case for a substantial and sustained European approach to cancer through public health measures and research.

## The Case for European Cancer Research

Europe needs both a strong commercial and a publicly funded (noncommercial) research base. In this article, we report the results of the European Cancer Research Funding Survey, which looked at noncommercial, public funding of cancer research. This survey has, for the first time, recognised the essential contribution that governments, charities, and European organisations make towards funding the broad church of research to find new ways to control and cure cancer. This funding, focused on the needs of patients with cancer rather than the commercial or economic advantage, is essential to deliver the myriad of solutions that cancer demands, from new strategies to prevent cancer, to therapies and improvements in patients' quality of life.

The public support of cancer research permits the necessary degree of variety and flexibility that is essential to preserving the diversity of cancer research [ 3]. This diversity in turn keeps the research possibilities sufficiently open to ensure that no good idea or discovery is lost. In order to support better evidence-based policy making in this important area, we undertook a pan-EU survey of the major public funders of cancer research.

## Our Survey Methods

Between July and December 2003, we sent letters to each of the general members of the European Cancer Research Managers Forum (ECRM; see http://www.ecrmforum.org), asking them to identify the sources of cancer research funding within their countries' boundaries. After the ECRM members had created an initial database of funding bodies for all countries within the EU, accession countries, and European free trade area (EFTA) associate and applicant states, all identified cancer research funding organisations were directly contacted. These funding bodies were asked to (1) provide their latest available figures for annual spending on direct cancer research, (2) provide a list of executive/board members, and (3) classify themselves as either governmental or charity organisations according to the enclosed guidelines.

We also asked respondents to report their annual spending on cancer research according to the seven main classifications of the common scientific outline (CSO), a classification system organised around seven broad areas of scientific interest in cancer research ( http://researchportfolio.cancer.gov/cso.html; Box 1). We chose to quantify annual spending by CSO because of its ability to compare and contrast research portfolios of public, nonprofit, and governmental research agencies using a validated and controlled vocabulary. Finally, respondents were provided with a list of all other identified funding bodies within their country for cross-validation. If a respondent organisation had a Web site, we examined the Web site for financial information, which we cross-referenced with the self-reported data. All information received and gathered was entered into an Access database.

At the beginning of the survey, guidelines were established to help with this quantification, and were followed through the data collection and data entry phases. If a funding organisation reported a range of spending on cancer research between two values, we always used the higher amount. In evaluating annual direct cancer research spending, we excluded educational grants, non–research staff salaries (other than research managers), physical plant improvements, and spending on advocacy and service delivery. CSO categories were only entered into the database if they were provided as a percent or monetary unit of the total spending. Any organization reporting spending in currencies other than the Euro had the reported amount of spending converted into Euros using the following Web site, http://www.xe.com; for replies received on or before 15 May 2004, conversions were made on that date. After this date, all currencies were converted within two days of receipt of the information.

Calculations such as gross domestic product (GDP) and GDP per capita were then made using ColdfusionMX ( http://www.macromedia.com/software/coldfusion), and were verified with Microsoft Excel, based upon data groomed from multiple sources.

At the end of the data collection phase of the survey, 123 of 139 identified funding organisations had reported back to the Secretariat, exceeding the 85% response rate set at the beginning of the survey to consider the work complete. One organization refused to provide any information other than contact details, and one other reported that there was no financial information publicly available.

Finally, we compared data from our EU survey with data on cancer research spending in the United States published by the US National Cancer Institute.

## Survey Results

### National variations in spending

**Figure 1:**
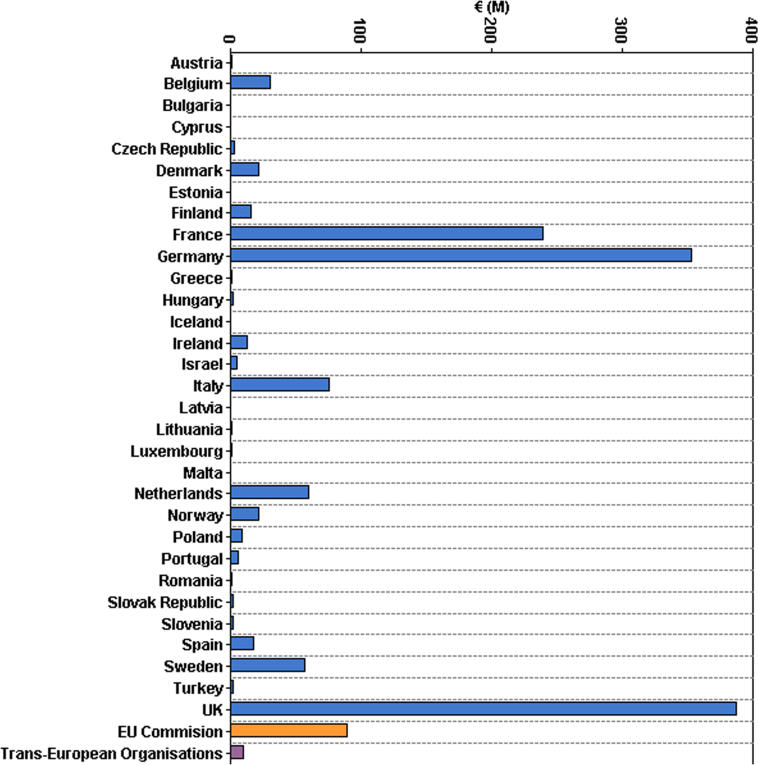
Direct Cancer Research Spending by Country, including European Commission and Trans-European Organisations

In our survey, we identified 139 noncommercial funding organisations that collectively spent €1.43 billion on cancer research for the year spanning 2002–2003. Absolute spending in 2002/2003 on cancer research varied widely across the EU, ranging from €388 million in the United Kingdom to €0 in Malta, with three countries spending greater than €100 million, nine greater than €10 million, and ten less than €1 million. Of all the countries in the survey, only Bulgaria failed to report their spending, and only Malta spent nothing on cancer research in 2002/2003 ( Figure 1).

### Spending by CSO in the EU and the US

**Figure 2:**
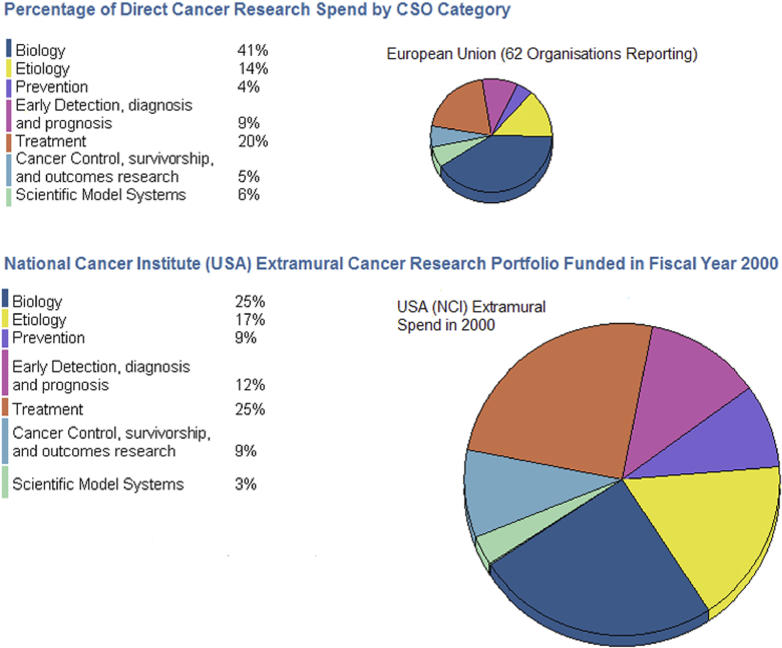
Percentage Spending by CSO: EU versus US The relative sizes of the two pie charts are proportional to the sizes of the two budgets.

The EU spends a greater proportion of its cancer research funding on cancer biology than does the US (41% compared with 25%). The US spends a greater proportion of its cancer research funding on research into prevention and treatment than does the EU (prevention, 9% in the US compared with 4% in the EU; treatment, 25% in the US compared with 20% in the EU) ( Figure 2). Data published by the US National Cancer Institute has been fully validated, whereas the EU uses self-reported, top-level CSO categories for 62% ( *n* = 74) of the organisations from which financial data was obtained. The size of the two pie charts in Figure 2 is representative of the sizes of the annual budgets: in 2002/2003, the US National Cancer Institute spent €3.60 billion, compared with the EU spending of €1.43 billion.

### Spending by charities versus governments

**Figure 3:**
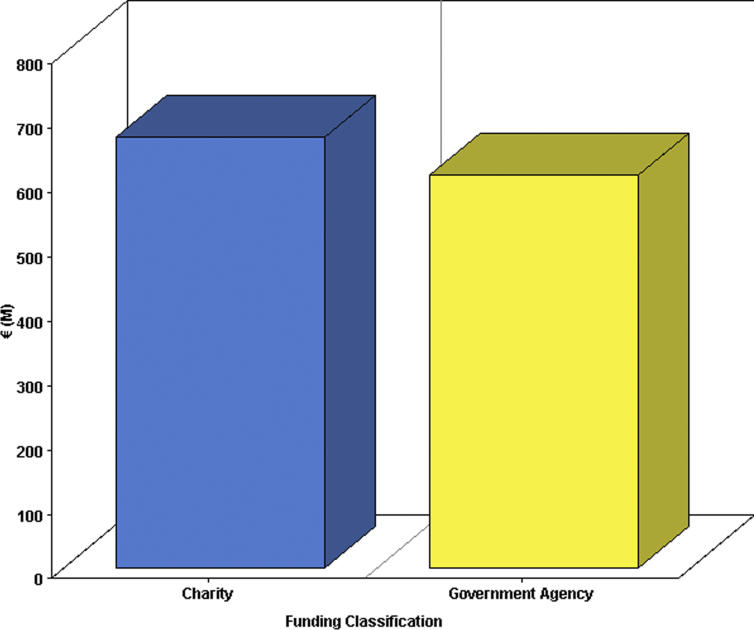
Direct Cancer Research Spending by Type of Funding Organisation

Our survey showed that just over 50% of noncommercial funding in the EU (including EFTA and associate states) is provided by the charitable sector, with 65 major charities across 23 countries contributing around €667.3 million to cancer research ( Figure 3). In comparison, there are 74 governmental sources of cancer research funding, spread across 28 countries, with a reported spending of €662.3 million in 2002/2003. It should be noted that these figures do not include contributions from organisations not contained within member state boundaries (Trans-European Organisations) and the EU Commission spending for 2002/2003.

For charities, the average amount spent per charity was €21.5 million, and the median spending was €400,000. Among governmental agencies, the average spent was €21.4 million, and their median spending was €1.9 million. In eight countries, there was no cancer research spending by charitable organisations, while in three countries, there was no spending by governments. In 11 countries, charities spent more on cancer research than did governments.

Of the 31 countries involved in this survey, six had more charities than governmental agencies, with ten having the same number for both types of funding organisations. The average number of charities per country was 2.1, and the average number of governmental agencies was 2.4.

### Spending by political grouping

**Figure 4:**
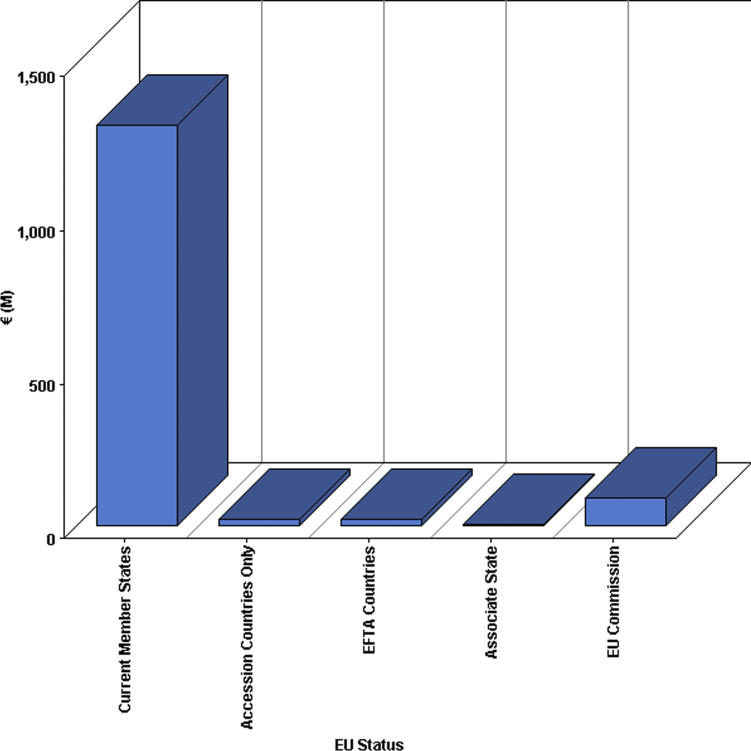
Funding By Political Grouping Current member states are all member states as of May 1, 2004. Accession countries are those member states which joined the EU in May 2004, or which are considered “applicant states”. EFTA countries include Iceland and Norway. Associate state is Israel.

Current member states ( *n* = 25) contributed €1.30 billion annually to noncommercial cancer research; the accession countries contributed only €21.3 million in 2002/2003, which was just 2% of the total annual amount ( Figure 4). The European Commission contributed €90 million, or just over 6%, of the identified total direct cancer research spending. However, this number is likely an underestimation, as cancer research is being supported by other, nondirect funding streams from the commission. However, to put this in perspective, the EU spends over €1 billion on tobacco subsidies every year.

### Total spending by the EU versus the US

**Figure 5:**
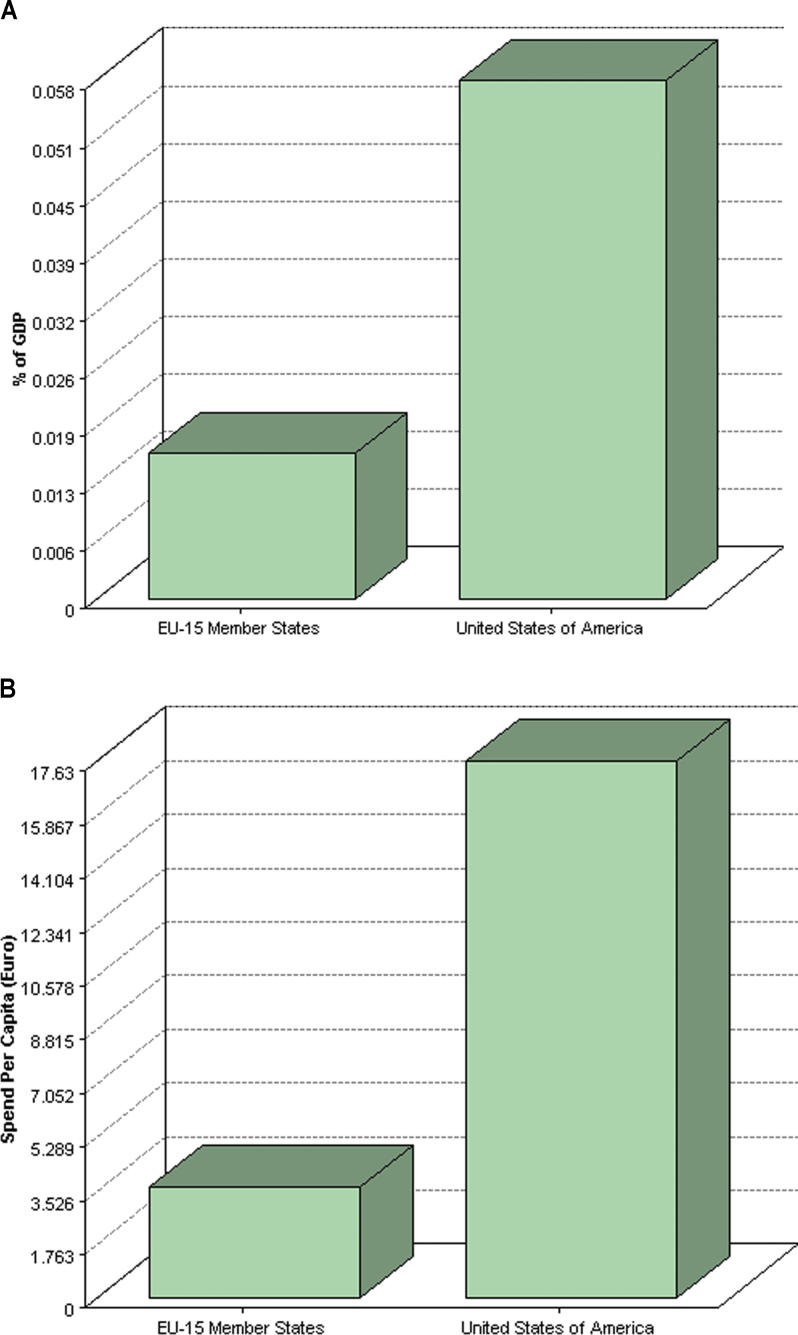
Comparison of Direct Cancer Research Spending between EU-15 Only and the US, as a Percentage of GDP and as Spending per Capita

The average per capita spent across the entire EU (including European Commission and Trans-European Organisation spending) was €2.56 (US$3.30), while the per capita spent in the US was €17.63 (US$22.76)—seven times greater. This gap is reduced to 5-fold if the US spending is compared with the spending of the 15 EU countries only ( Figure 5). Average cancer research spending as a percentage of GDP across the EU was 0.0152%, and the median was 0.0056%. As a percentage of GDP, the US spent four times as much as the average across the entire European survey; this difference remained the same when the US percentage was compared with the percentage spending by the 15 EU member states.

## Discussion

Cancer research plays a dominant role in two of the EU's political objectives—advancing knowledge in health and advancing biotechnology. The European Commission has made strengthening European research a major objective. At the Lisbon European Council of March 2000, the European Research Area was launched—this is an initiative to increase the funding and productivity of EU research and development. Within its framework, the ambitious objective was set of increasing research spending to 3% of the EU's GDP by 2010 [ 4]. During this period of the Sixth Framework Programme (FP6)—the financial instrument to help make the European Research Area a reality ( http://www.cordis.lu/fp6/whatisfp6.htm)—the European Commission placed increased emphasis on further political commitment by member states to research funding and the establishment of European frameworks through commission funding tools. Examples of these tools are “networks of excellence”, designed to strengthen scientific and technological excellence on a particular research topic ( http://europa.eu.int/comm/research/fp6/pdf/noe_120503final.pdf), and “integrated projects”, an “instrument to support objective-driven research” ( http://europa.eu.int/comm/research/fp6/pdf/ip_provisions_120503final.pdf).

### Insufficient funding of cancer research in the EU

However, despite the recognition that greater investment by the private sector requires more EU research to be funded, the FP6 has been insufficient. Only one in five fundable proposals has been supported, of which only 50% of projects judged to be of a very high quality were financed [ 5]. Our survey shows that the FP6 has also been inadequate to support the current research needs of cancer at a trans-European level.

Furthermore, the wide variation in available funds for cancer research at a member-state level inevitably means that the EU funding is not complementary but subsidising. Such national disparities in cancer research funding are particularly pronounced in the ten accession countries. Given the need for the whole of the enlarged EU to engage in the Lisbon agenda, the evidence gathered by this survey suggests that urgent and specific measures are needed to realise the potential of these member states. While many recommendations have been made following the recent review of the framework programmes, few of these will have any meaningful impact upon cancer research without a substantial increase in EU funding of cancer research [ 6].

The results of our survey are highly relevant to two of the major objectives of the EU's science and technology policy—making Europe more attractive to the best researchers and improving the coordination of national research programmes. Cancer research is critically dependent on recruiting and retaining the brightest minds across a very wide spectrum of scientific and clinical disciplines. Stimulating research funding and creating infrastructure or technology platforms are irrelevant without the researchers to use them. Cancer research, as with most biomedical research, needs concerted efforts to prevent “brain drain”, as well as recruit the ablest students from both traditional and nontraditional scientific disciplines [ 7]. Funding for cancer research is concentrated at member-state level, and thus by implication so too is most of the funding for clinical and nonclinical fellowships. This finding, along with data in the public domain showing that a number of trans-European organisations (such as the European Organisation for the Research and Treatment of Cancer and the European School of Oncology) also support a variety of fellowships, presents an opportunity to review the availability of fellowship support, with a view to creating additional targeted funding in this area through the Seventh Framework Programme (FP7)[ 8]. FP7 is the EU's chief instrument for funding scientific research and technological development over the period of 2007–2013 ( http://www.cordis.lu/fp7) [ 8].

FP6 has seen resources, through the European Research Area Network, to network national and regional programmes and increase the mutual opening up of national and regional programmes. The prevailing policy view is that research activities are duplicative and fragmented. However, our findings suggest otherwise for cancer research. Although 138 major funders of cancer research were identified, over 80% of the funding is accounted for by 25 organisations. Furthermore, we found no evidence to support any duplication of effort in cancer research, although such an assessment was not one of the primary aims of this survey.

Even if the 3% of GDP for public and private research investment is not met by 2010, as is now predicted, the framework programme in its next iteration (FP7) could nevertheless substantially improve cancer research and its applications to new therapeutics and diagnostics. It could make such improvements if, and only if, there is a substantial increase in EU spending (both at a central level and at a member-state level) directed specifically at clinical cancer research [ 9]. Furthermore, this increased spending at EU level must be tied to a more transparent approach to funding truly trans-EU cancer research.

### The gap between US and EU funding

It has previously been acknowledged that the EU fails to match the private or public funding levels of the US in cancer research and development, but just how large the gap has been for noncommercial (public) funding has not been appreciated until now [ 10]. A survey of cancer research funders, similar to our own survey, has been undertaken in the US. In 1999 the National Cancer Policy Board conducted a survey of federal and nonfederal sources of cancer research funding [ 11]. The board found, for the fiscal year 1996/1997, that the total amount spent on cancer research funding was US$5.165 billion. The three major contributors were (1) federal funding, US$3.060 billion (almost entirely from National Cancer Institute); (2) industry funding, US$1.6 billion; and (3) funding by nonprofit organisations (e.g., Howard Hughes Medical Institute, American Cancer Society, Komen Foundation), US$305 million.

This important US survey noted the growing contribution of the nonprofit philanthropic sector. The National Cancer Policy Board estimated that each state also contributed about US$200 million per year. This survey found a multiplicity of noncommercial cancer research funding streams within the domains of federal and nonprofit catagories: 20 National Institutes of Health, four additional departments of the Health and Human Services Agencies, a number of additional federal agencies, and over eight major foundations and nonprofit organisations, in addition to state funding directly to cancer centres, e.g., Texas/M. D. Anderson described above. In the years following this major policy review, US cancer research funding grew at a substantial rate. In the fiscal year 2002, the total National Cancer Institute obligations alone came to US$4.192 billion (this includes both intramural and extramural funds), representing nearly a doubling of funds since 1997 [ 12].

For a number of reasons, our own survey may have underestimated the size of the gap between EU and US funding of cancer research. First, we excluded the accession countries in our comparison between the EU and the US. Adding these countries to the calculations of spending as a percentage of GDP or per capita would significantly increase the denominator for the EU without any significant addition to the numerator. Second, although there are validated 2002/2003 spending figures for the NCI, American Cancer Society, and Department of Defense, all other noncommercial US data are drawn from the 1997 National Cancer Policy Board review. Although the significant noncommercial funders have been updated, the figure for overall US spending will therefore be an underestimate. While these important caveats magnify the funding gap, they do not change the basic message from this survey: the EU is massively behind the US in its support of noncommercial cancer research. The underlying data to this overall comparison indicates that the problem lies both with a lack of central EU funding and with many member states failing to adequately support cancer researchers in their countries.

Our finding of a difference in cancer research spending between the US and the EU is also consistent with the generally widening gap in spending on research and development between the US and the EU, which in 2002 stood at more than €124 billion (US$160 billion) [ 13]. If the funding of noncommercial cancer research is also widening, then this has major implications both for the ability of the EU to reverse the emigration of cancer researchers to the US and for the EU's overall commercial attractiveness. With such a close correlation between research activity and high-quality service delivery, spending too little on research is also likely to have an effect, ultimately on the overall care of patients with cancer.

A strong EU noncommercial cancer research sector is an integral promoter of cancer biotechnology. Despite the underperforming European biotechnology sector, the oncology market worldwide has witnessed remarkable growth and is poised for even greater growth [ 14]. However, the high attrition rate and increasing complexity of developing and conducting research into new anticancer agents, as well as nondrug interventions, diagnostics, and predictive and prognostic markers, requires strong pan-European networks linking major centres. The critical issue is the fostering of a European cancer research noncommercial oligopoly that can tackle the major challenges from a variety of approaches. The preliminary data indicating that across the EU less clinical research is funded as a proportion of total spending is worrisome and needs to be urgently addressed.

## Conclusion

While the utility of assessing cancer research outputs is widely recognised, no studies have focused on the funding bodies as a unit of evaluation. By identifying the major organisational funders of cancer research across Europe the European Cancer Research Funding Survey will further enable high-resolution studies aimed at evaluating the research activity and impact of funding agencies, particularly those in the public sector [ 15].

**Box 1. The Seven Components of the CSO**

- Biology
- Etiology (causes of disease)
- Prevention
- Early detection, diagnosis, and prognosis
- Treatment
- Cancer control, survivorship, and outcomes research
- Scientific model systems (e.g., computer simulation, cell culture, and organ, tissue, and animal models)

(Data from http://researchportfolio.cancer.gov/cso.html)

## Abbreviations

CSO: common scientific outline
ECRM: European Cancer Research Managers Forum
EFTA: European free trade area
EU: European Union
FP6: Sixth Framework Programme
FP7: Seventh Framework Programme
GDP: gross domestic product
